# Carnosinium bromide

**DOI:** 10.1107/S2414314626007261

**Published:** 2026-07-21

**Authors:** Fiona Hamilton, William T. A. Harrison

**Affiliations:** a Department of Chemistry, University of Aberdeen, Meston Walk, Aberdeen AB24 3UE, Scotland, United Kingdom; Universitat de les Illes Balears, Palma de Mallorca, Spain

**Keywords:** crystal structure, carnosine, dipeptide, salt

## Abstract

The title salt, C_9_H_15_N_4_O_3_^+^·Br^−^ [systematic name: 5-[(2*S*)-2-(3-azan­ium­yl­pro­pan­amido)-2-car­box­yl­ato­ethyl]-1*H*-imid­azol-3-ium bro­mide], contains four cations and four anions in the asymmetric unit. The dipeptide is a ’cationic zwitterion’ with two localized positive charges and one delocalized negative charge. Two of the cations adopt an *anti* conformation for the non-hydrogen atoms of the –C(=O)—CH_2_—CH_2_—NH_3_^+^ grouping and two adopt a *gauche* conformation. In the extended structure, numerous N—H⋯O, N—H⋯(O,O) and N—H⋯Br hydrogen bonds link the components into (010) sheets and the structure is consolidated by weak C—H⋯O and C—H⋯Br inter­actions.

## Abstract 2

The asymmetric unit of the title salt contains four cations and four anions. The dipeptide is a ’cationic zwitterion’ with two localized positive charges and one delocalized negative charge. Two of the cations adopt an *anti* conformation for the non-hydrogen atoms of the –C(=O)—CH_2_—CH_2_—NH_3_^+^ grouping and two adopt a *gauche* conformation. In the extended structure, numerous N—H⋯O, N—H⋯(O,O) and N—H⋯Br hydrogen bonds link the components into (010) sheets and the structure is consolidated by weak C—H⋯O and C—H⋯Br inter­actions.

## Graphical abstract

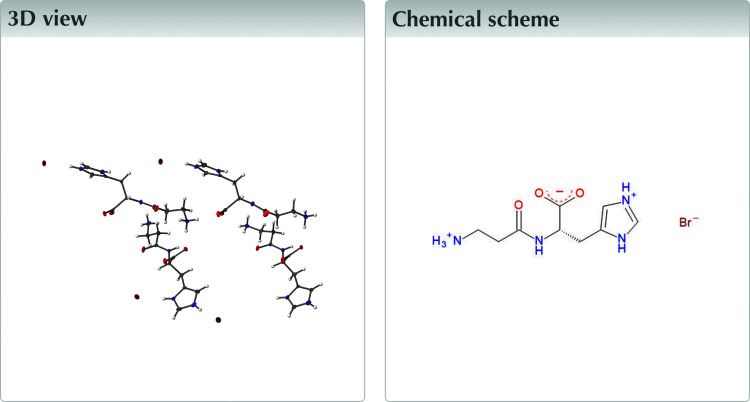

## Structure description

Carnosine [β-alanyl-l-histidine; C_9_H_14_N_4_O_3_; systematic name (2*S*)-2-(3-amino­propanamido)-3-(3*H*-imidazol-4-yl)propanoic acid] is a naturally occurring dipeptide made up from β-alanine and histidine, which occurs in the muscle and brain tissue of higher animals (Jukić *et al.*, 2021 ▸). The crystal structure of carnosine (Barrans *et al.*, 1976 ▸) with Cambridge Structural Database (Groom *et al.*, 2016 ▸) refcode BALHIS shows it to occur as a zwitterion (nominal proton transfer from the histidine carb­oxy­lic acid group to the β-alanine terminal –NH_2_ group) in the solid state. The crystal structures of several salts of carnosine with natural (di)carb­oxy­lic acids were reported by Shemchuk *et al.* (2017 ▸), including carnosinium glycolate, C_9_H_15_N_4_O_3_^+^·C_2_H_3_O_3_^−^ (HAXYEN), carnosinium salicylate monohydrate, C_9_H_15_N_4_O_3_^+^·C_7_H_5_O_3_^−^·H_2_O (HAXXEM) and carnosinium hydrogen azelate, C_9_H_15_N_4_O_3_^+^·C_9_H_15_O_4_^−^ (HAXYAJ). As part of our studies in this area, we now describe the synthesis and structure of the title salt, C_9_H_15_N_4_O_3_^+^·Br^−^ (**I**), also called carnosine hydro­bromide. The chloride salt, C_9_H_15_N_4_O_3_^+^·Cl^−^, is probably isostuctural with (**I**) but unresolvable twinning precluded detailed structural analysis.

**Figure 1:**
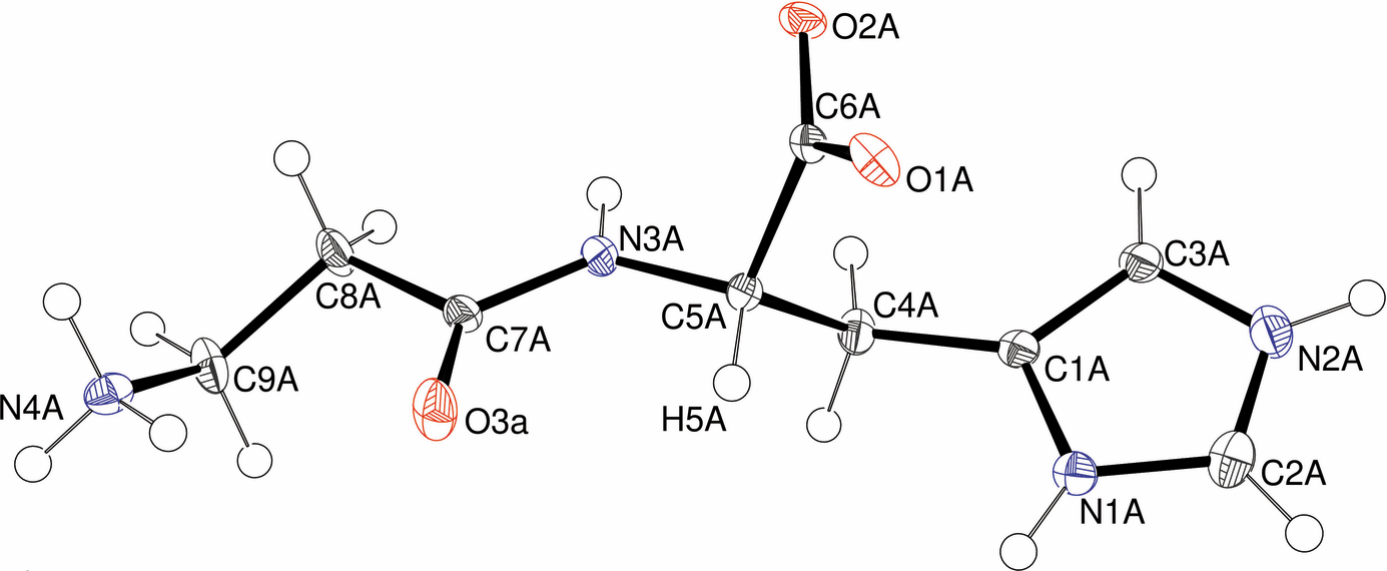
The *A*-suffix cation in the asymmetric unit of (**I**) showing 50% displace­ment ellipsoids.

The asymmetric-unit of (**I**), which crystallizes in the monoclinic space group *P*2_1_ with β ≃ 90°, contains four C_9_H_15_N_4_O_3_^+^ cations and four bromide anions. Equivalent atoms in the cations have been assigned the suffixes *A*, *B*, *C* and *D* (Fig. 1 ▸). For each cation, which might be described as a ’cationic zwitterion,’ a formal positive charge resides on the terminal alanyl –N4H_3_^+^ moiety and the N2H^+^ grouping of the imidazolinium ring and a net negative charge is delocalized over the histidine –C6/O1/O2 carboxyl­ate group (see scheme).

**Figure 2:**
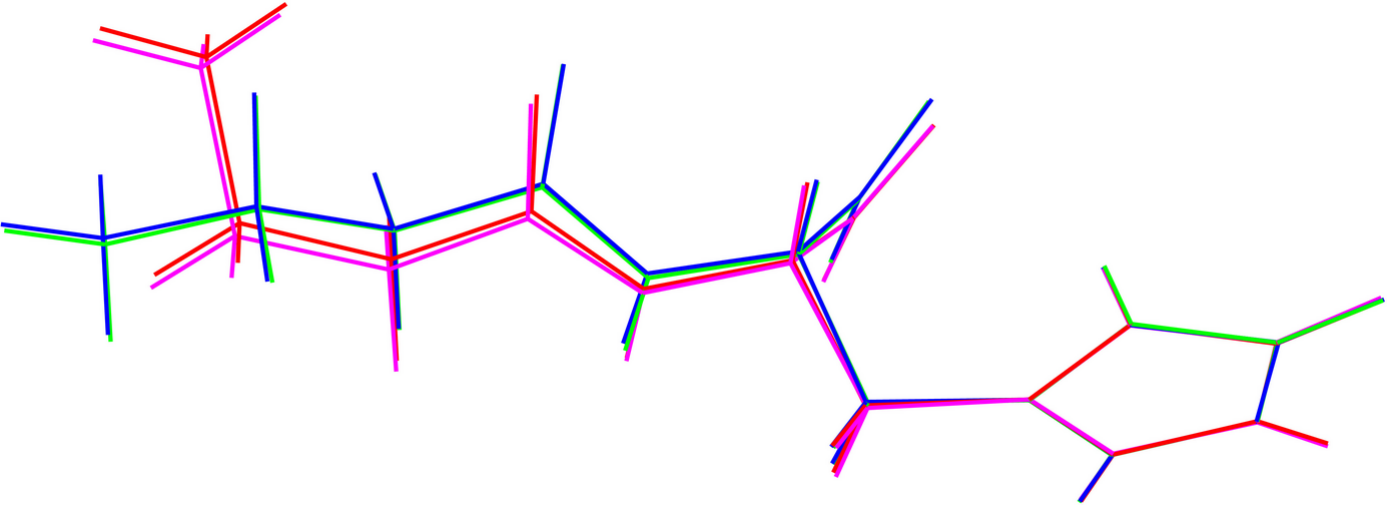
Overlay plot of the four distinct cations in (**I**): *A* red, *B* blue, *C* green and *D* purple.

The absolute structure of (**I**) is well defined based on refinement of the Flack absolute structure parameter (Parsons *et al.*, 2013 ▸) to −0.028 (5) and, as expected for l-carnosine, the stereogenic (chiral) carbon atom C5 from the histidine residue has an *S* configuration in each cation (there is no stereogenic centre in a β-amino acid). An overlay plot (Fig. 2 ▸) generated with *Qmol* (Gans & Shalloway, 2001 ▸) shows that the cations have similar but not identical conformations with the major difference being the relative orientations of atoms C7 and N4 about the C8—C9 bond of the β-alanyl chain, being *gauche* for the *A* [torsion angle = 69.9 (8)°] and *D* [68.2 (8)°] cations and *anti* for *B* [174.7 (6)°] and *C* [176.5 (5)°]. The dihedral angles between the C6/O1/O2 carboxyl­ate group and the C1–C3/N1/N2 imidazole ring plane are 50.1 (6), 55.4 (6), 55.7 (6) and 50.0 (6)° for cations *A*, *B*, *C* and *D*, respectively, suggesting some correlation with the different torsion angles mentioned in the previous sentence. The C2—N2—C3 bond angle in the imidazole ring at the protonated N atom (mean value for the four cations = 109.3°) is expanded by about 4° in (**I**) compared to the equivalent unprotonated bond angle in carnosine (Barrans *et al.*, 1976 ▸). Otherwise, the geometric data for the four cations in (**I**) are unexceptional and broadly similar to each other; selected torsion angles for these and some related structures are presented in Table 1 ▸.

These data show that the C_9_H_14_N_4_O_3_ neutral zwitterion and the C_9_H_15_N_4_O_3_^+^ cationic zwitterion can adopt distinctly different conformations in the solid state, presumably to optimize the packing. For the ions listed, five show an *anti* conformation for the C7—C8—C9—N4 grouping (our atom labels) and four are *gauche* and it may be noted that one of the two distinct cations in HAXYAJ has a *gauche* conformation and the other is *anti*. The C4—C5 bond shows even more conformational flexibility in the solid-state, with C1 being *anti* to N3, C6 or even H5 in different structures. The conformational flexibility of carnosine in aqueous solution has been studied by spectroscopic methods (Barrans *et al.*, 1976 ▸).

**Figure 3:**
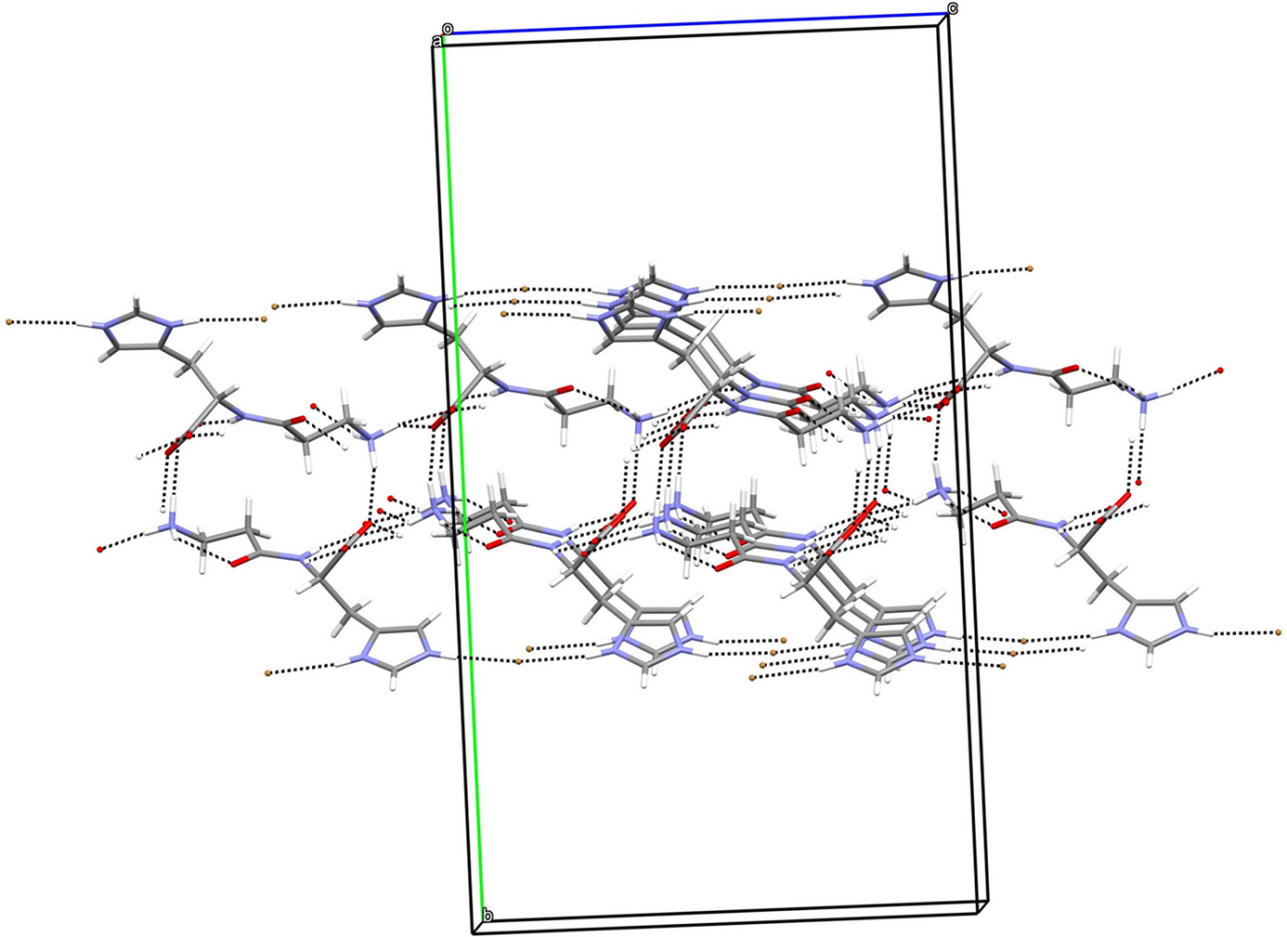
The unit-cell packing in (**I**) viewed approximately down [100] with hydrogen bonds shown as black dashed lines. Note that the N—H⋯Br hydrogen bonds lie on the outer faces of the (010) sheets.

In the extended structure of (**I**), numerous N—H⋯O, N—H⋯(O,O) and N—H⋯Br hydrogen bonds (Table 2 ▸) link the components into (010) sheets (Fig. 3 ▸). For each cation, the N1H and N2H^+^ moieties of the imidazole ring form N—H⋯Br hydrogen bonds, which lie on the outer surfaces of the sheets, while the amide N3H grouping and the protonated terminal –N4H_3_^+^ group participate in N—H⋯O or bifurcated N—H⋯(O,O) hydrogen bonds. Considered by themselves, the former bonds generate [001] chains, while the connectivity of the latter is two-dimensional. Many weak C—H⋯Br and C—H⋯O links are also present - some of these reinforce the (010) sheets and others link them into a three-dimensional supra­molecular network.

## Synthesis and crystallization

The title compound was prepared by mixing 1.1 g of l-carnosine, 5 ml of 1.0 *M* HBr solution and 20 ml of water (carnosine:Br molar ratio ≃ 1:1), which resulted in a colourless solution. The solution was left in a Petri dish at room temperature and colourless rods and blocks of (**I**) formed as the water evaporated over a few days.

## Refinement

Crystal data, data collection and structure refinement details are summarized in Table 3 ▸. The H atoms were located geometrically (N—H = 0.88–0.91 Å, C—H = 0.95–0.99 Å) and refined as riding atoms. The –NH_3_ groups were allowed to rotate, but not to tip, about the bond to the adjacent C atom to best fit the electron density. The constraint *U*_iso_(H) = 1.2*U*_eq_(carrier) was applied in all cases. RIGU and ISOR cards in *SHELXL* (Sheldrick, 2015*b* ▸) were applied to ensure that the anisotropic displacement parameters for bonded atoms were physically reasonable. The crystal chosen for data collection was found to be twinned, which may correlate with the β angle being very close to 90°. Non-merohedral twin (HKLF 5 in *SHELXL*) refinements were not successful and a two-component (HKLF 4) *pseudo*-merohedral twin refinement with a twinning matrix −1 0 0 / 0 − 1 0 / 0 0 1 was preferred but this has led to a poor data completion percentage where some closely overlapping reflections from different domains were excluded. The refined domain ratio is 0.7892 (19): 0.2108 (19).

## Supplementary Material

Crystal structure: contains datablock(s) I, global. DOI: 10.1107/S2414314626007261/tk4128sup1.cif

Structure factors: contains datablock(s) I. DOI: 10.1107/S2414314626007261/tk4128Isup2.hkl

CCDC reference: 2573338

Additional supporting information:  crystallographic information; 3D view; checkCIF report

## Figures and Tables

**Table 1:** Selected torsion angles (°) for the cations in (**I**) and related phases φ is the C4—C5—N3—C7 torsion angle; ψ is the C1—C4—C5—N3 torsion angle; ω is the C5—N3—C7—C8 torsion angle; χ is the C1—C4—C5—C6 torsion angle; ρ is the C7—C8—C9—N4 torsion angle; ξ is the C1—C4—C5—H5 torsion angle (our atom-labelling scheme; see Fig. 1 ▸).

Cation	φ	ψ	ω	χ	ρ	ξ
(**I**)*A*	131.0 (7)	171.1 (6)	176.9 (6)	53.0 (8)	69.9 (8)	–68
(**I**)*B*	135.4 (6)	173.6 (6)	–179.0 (5)	52.1 (8)	174.7 (6)	–68
(**I**)*C*	137.8 (6)	173.7 (6)	–179.9 (6)	53.4 (7)	176.5 (5)	–67
(**I**)*D*	129.1 (7)	170.9 (6)	177.7 (6)	50.8 (7)	68.2 (8)	–69
BALHIS^*a*,^^*b*^	146.7	–178.5	174.4	61.2	177.1	–61
HAXYEN^*a*^	76.5	68.5	175.2	–51.9	–176.2	–174
HAXXEM^*a*^	157.6	–69.7	157.6	169.9	–68.0	–48
HAXYAJ(*A*)	163.8	173.5	173.3	52.0	171.9	–64
HAXYAJ(*B*)	158.5	171.8	168.4	50.1	64.3	–72

Notes: (*a*) The deposited atomic coordinates for BALHIS, HAXYEN and HAXXEM correspond to the *R*-enanti­omer of carnosine and they have been inverted to calculate the torsion angles listed here. (*b*) BALHIS is a neutral zwitterion.

**Table 2:** Hydrogen-bond geometry (Å, °)

*D*—H⋯*A*	*D*—H	H⋯*A*	*D*⋯*A*	*D*—H⋯*A*
N1*A*—H1*A*⋯Br4^i^	0.88	2.39	3.242 (7)	162
N2*A*—H2*A*1⋯Br2^ii^	0.88	2.33	3.205 (6)	171
N3*A*—H3*A*1⋯O1*A*^i^	0.88	2.04	2.918 (9)	172
N4*A*—H4*A*3⋯O1*B*^iii^	0.91	1.88	2.771 (7)	167
N4*A*—H4*A*4⋯O1*C*^i^	0.91	1.84	2.744 (7)	171
N4*A*—H4*A*5⋯O3*A*	0.91	2.23	2.887 (7)	129
N4*A*—H4*A*5⋯O2*B*^i^	0.91	2.36	2.875 (8)	116
C2*A*—H2*A*⋯Br1^iv^	0.95	2.71	3.651 (8)	170
C4*A*—H4*A*1⋯Br4^iii^	0.99	2.77	3.678 (6)	153
C9*A*—H9*A*1⋯O3*A*^i^	0.99	2.54	3.156 (10)	120
N1*B*—H1*B*⋯Br2	0.88	2.40	3.261 (7)	167
N2*B*—H2*B*1⋯Br4	0.88	2.29	3.167 (6)	173
N3*B*—H3*B*1⋯O1*B*^i^	0.88	2.04	2.907 (8)	170
N4*B*—H4*B*3⋯O1*A*^v^	0.91	1.86	2.764 (7)	171
N4*B*—H4*B*4⋯O3*B*^i^	0.91	1.96	2.798 (8)	153
N4*B*—H4*B*5⋯O2*D*^i^	0.91	1.85	2.744 (7)	169
C2*B*—H2*B*⋯O3*A*^vi^	0.95	2.58	3.425 (10)	149
C3*B*—H3*B*⋯Br3^iv^	0.95	2.64	3.587 (8)	171
C4*B*—H4*B*1⋯Br2^i^	0.99	2.93	3.836 (6)	152
C5*B*—H5*B*⋯Br2	1.00	3.12	4.046 (7)	155
C8*B*—H8*B*2⋯O3*B*^i^	0.99	2.66	3.341 (9)	127
C9*B*—H9*B*2⋯O2*A*^vii^	0.99	2.38	3.307 (9)	156
N1*C*—H1*C*⋯Br1^ii^	0.88	2.39	3.251 (6)	165
N2*C*—H2*C*1⋯Br3	0.88	2.30	3.165 (7)	168
N3*C*—H3*C*1⋯O2*C*^i^	0.88	2.03	2.905 (7)	171
N4*C*—H4*C*3⋯O1*D*^viii^	0.91	1.85	2.760 (8)	176
N4*C*—H4*C*4⋯O3*C*^i^	0.91	1.96	2.808 (7)	154
N4*C*—H4*C*5⋯O2*A*	0.91	1.84	2.747 (7)	176
C2*C*—H2*C*⋯O3*D*^i^	0.95	2.58	3.422 (9)	148
C3*C*—H3*C*⋯Br2^ix^	0.95	2.64	3.583 (8)	172
C4*C*—H4*C*1⋯Br1^viii^	0.99	2.85	3.776 (8)	155
C9*C*—H9*C*2⋯O2*D*^ii^	0.99	2.37	3.300 (8)	156
N1*D*—H1*D*⋯Br3^vi^	0.88	2.38	3.234 (6)	165
N2*D*—H2*D*1⋯Br1	0.88	2.33	3.204 (7)	173
N3*D*—H3*D*1⋯O1*D*^i^	0.88	2.05	2.926 (7)	172
N4*D*—H4*D*3⋯O2*C*	0.91	1.88	2.772 (8)	167
N4*D*—H4*D*4⋯O2*B*	0.91	1.85	2.751 (7)	171
N4*D*—H4*D*5⋯O1*C*^vi^	0.91	2.36	2.875 (7)	116
N4*D*—H4*D*5⋯O3*D*	0.91	2.22	2.871 (8)	128
C3*D*—H3*D*⋯Br4^x^	0.95	2.72	3.662 (8)	170
C4*D*—H4*D*1⋯Br3	0.99	2.86	3.746 (7)	149
C9*D*—H9*D*1⋯O3*D*^i^	0.99	2.53	3.146 (8)	120

Symmetry codes: (i) 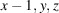; (ii) 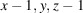; (iii) 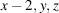; (iv) 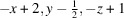; (v) 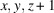; (vi) 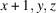; (vii) 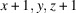; (viii) 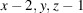; (ix) 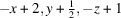; (x) 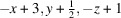.

**Table 3:** Experimental details

Crystal data	
Chemical formula	C_9_H_15_N_4_O_3_^+^·Br^−^
*M*_r_	307.16
Crystal system, space group	Monoclinic, *P*2_1_
Temperature (K)	100
*a*, *b*, *c* (Å)	5.46611 (13), 28.6332 (5), 16.2890 (4)
β (°)	90.012 (2)
*V* (Å^3^)	2549.43 (10)
*Z*	8
Radiation type	Mo *K*α
μ (mm^−1^)	3.23
Crystal size (mm)	0.19 × 0.05 × 0.04

Data collection	
Diffractometer	Rigaku CCD
Absorption correction	Multi-scan (*CrysAlis PRO*; Rigaku OD, 2015 ▸)
*T*_min_, *T*_max_	0.791, 1.000
No. of measured, independent and observed [*I* > 2σ(*I*)] reflections	18071, 7858, 7654
*R*_int_	0.019
(sin θ/λ)_max_ (Å^−1^)	0.649

Refinement	
*R*[*F*^2^ > 2σ(*F*^2^)], *wR*(*F*^2^), *S*	0.035, 0.092, 1.06
No. of reflections	7858
No. of parameters	618
No. of restraints	445
H-atom treatment	H-atom parameters constrained
Δρ_max_, Δρ_min_ (e Å^−3^)	0.85, −0.42
Absolute structure	Flack *x* determined using 2376 quotients [(*I*^+^)−(*I*^−^)]/[(*I*^+^)+(*I*^−^)] (Parsons *et al.*, 2013 ▸)
Absolute structure parameter	−0.018 (5)

Computer programs: *CrysAlis PRO* (Rigaku OD, 2015 ▸), *SHELXT* (Sheldrick, 2015*a* ▸), *SHELXL2019/2* (Sheldrick, 2015*b* ▸), *ORTEP-3 for Windows* (Farrugia, 2012 ▸) and *publCIF* (Westrip, 2010 ▸).

## full crystallographic data

### 5-[(2S)-2-(3-Azaniumylpropanamido)-2-carboxylatoethyl]-1H-imidazol-3-ium bromide Crystal data

**Table:**

C_9_H_15_N_4_O_3_^+^·Br^−^	*F*(000) = 1248
*M**_r_* = 307.16	*D*_x_ = 1.601 Mg m^−^^3^
Monoclinic, *P*2_1_	Mo *K*α radiation, λ = 0.71073 Å
*a* = 5.46611 (13) Å	Cell parameters from 11802 reflections
*b* = 28.6332 (5) Å	θ = 2.4–29.5°
*c* = 16.2890 (4) Å	µ = 3.23 mm^−^^1^
β = 90.012 (2)°	*T* = 100 K
*V* = 2549.43 (10) Å^3^	Block, colourless
*Z* = 8	0.19 × 0.05 × 0.04 mm

### 5-[(2S)-2-(3-Azaniumylpropanamido)-2-carboxylatoethyl]-1H-imidazol-3-ium bromide Data collection

**Table:**

Rigaku CCD diffractometer	7858 independent reflections
Radiation source: fine-focus sealed X-ray tube	7654 reflections with *I* > 2σ(*I*)
Graphite monochromator	*R*_int_ = 0.019
ω scans	θ_max_ = 27.5°, θ_min_ = 2.5°
Absorption correction: multi-scan (CrysAlisPro; Rigaku OD, 2015)	*h* = −7→7
*T*_min_ = 0.791, *T*_max_ = 1.000	*k* = −37→36
18071 measured reflections	*l* = −20→21

### 5-[(2S)-2-(3-Azaniumylpropanamido)-2-carboxylatoethyl]-1H-imidazol-3-ium bromide Refinement

**Table:**

Refinement on *F*^2^	Hydrogen site location: inferred from neighbouring sites
Least-squares matrix: full	H-atom parameters constrained
*R*[*F*^2^ > 2σ(*F*^2^)] = 0.035	*w* = 1/[σ^2^(*F*_o_^2^) + (0.0529*P*)^2^ + 3.5341*P*] where *P* = (*F*_o_^2^ + 2*F*_c_^2^)/3
*wR*(*F*^2^) = 0.092	(Δ/σ)_max_ = 0.001
*S* = 1.06	Δρ_max_ = 0.85 e Å^−^^3^
7858 reflections	Δρ_min_ = −0.41 e Å^−^^3^
618 parameters	Absolute structure: Flack *x* determined using 2376 quotients [(*I*^+^)-(*I*^-^)]/[(*I*^+^)+(*I*^-^)](Parsons *et al.*, 2013)
445 restraints	Absolute structure parameter: −0.018 (5)
Primary atom site location: structure-invariant direct methods	

### 5-[(2S)-2-(3-Azaniumylpropanamido)-2-carboxylatoethyl]-1H-imidazol-3-ium bromide Special details

**Table:**

Geometry. All esds (except the esd in the dihedral angle between two l.s. planes) are estimated using the full covariance matrix. The cell esds are taken into account individually in the estimation of esds in distances, angles and torsion angles; correlations between esds in cell parameters are only used when they are defined by crystal symmetry. An approximate (isotropic) treatment of cell esds is used for estimating esds involving l.s. planes.
Refinement. Refined as a 2-component twin.

### 5-[(2S)-2-(3-Azaniumylpropanamido)-2-carboxylatoethyl]-1H-imidazol-3-ium bromide Fractional atomic coordinates and isotropic or equivalent isotropic displacement parameters (Å^2^)

**Table:**

	*x*	*y*	*z*	*U*_iso_*/*U*_eq_	
C1A	−0.3846 (13)	0.3281 (2)	−0.0699 (4)	0.0124 (13)	
C2A	−0.0596 (13)	0.2872 (3)	−0.1059 (5)	0.0174 (15)	
H2A	0.082943	0.268239	−0.103299	0.021*	
C3A	−0.3642 (14)	0.3289 (2)	−0.1533 (5)	0.0157 (15)	
H3A	−0.470595	0.344238	−0.190686	0.019*	
C4A	−0.5716 (13)	0.3490 (2)	−0.0142 (5)	0.0149 (15)	
H4A1	−0.636412	0.324211	0.022169	0.018*	
H4A2	−0.709365	0.360817	−0.047756	0.018*	
C5A	−0.4734 (13)	0.3892 (2)	0.0394 (4)	0.0137 (14)	
H5A	−0.353554	0.377286	0.080723	0.016*	
C6A	−0.3513 (13)	0.4250 (2)	−0.0182 (4)	0.0116 (12)	
C7A	−0.6965 (13)	0.4198 (2)	0.1600 (4)	0.0146 (13)	
C8A	−0.9305 (14)	0.4446 (3)	0.1870 (4)	0.0182 (14)	
H8A1	−0.907562	0.478773	0.181602	0.022*	
H8A2	−1.066138	0.435311	0.150213	0.022*	
C9A	−0.9999 (14)	0.4332 (2)	0.2756 (4)	0.0188 (15)	
H9A1	−1.167253	0.444823	0.286585	0.023*	
H9A2	−1.000967	0.398846	0.282924	0.023*	
N1A	−0.1897 (11)	0.3016 (2)	−0.0417 (4)	0.0139 (12)	
H1A	−0.157361	0.295366	0.010027	0.017*	
N2A	−0.1589 (12)	0.3031 (2)	−0.1729 (4)	0.0193 (14)	
H2A1	−0.103855	0.298192	−0.222880	0.023*	
N3A	−0.6839 (11)	0.41061 (19)	0.0801 (4)	0.0143 (12)	
H3A1	−0.811156	0.417885	0.049550	0.017*	
N4A	−0.8298 (10)	0.45406 (18)	0.3346 (3)	0.0151 (12)	
H4A3	−0.871740	0.445109	0.386372	0.023*	
H4A4	−0.837230	0.485733	0.330795	0.023*	
H4A5	−0.674946	0.444306	0.323460	0.023*	
O1A	−0.1215 (10)	0.42585 (18)	−0.0190 (3)	0.0182 (11)	
O2A	−0.4879 (9)	0.44842 (16)	−0.0632 (3)	0.0151 (10)	
O3A	−0.5306 (10)	0.41137 (18)	0.2094 (3)	0.0213 (11)	
C1B	0.6602 (13)	0.3296 (2)	0.4277 (4)	0.0133 (14)	
C2B	0.6724 (15)	0.3285 (2)	0.3448 (5)	0.0183 (15)	
H2B	0.562893	0.343211	0.307660	0.022*	
C3B	0.9825 (14)	0.2865 (3)	0.3922 (5)	0.0197 (16)	
H3B	1.123696	0.267263	0.395021	0.024*	
C4B	0.4841 (13)	0.3530 (2)	0.4843 (4)	0.0136 (14)	
H4B1	0.412884	0.329230	0.521459	0.016*	
H4B2	0.348939	0.366524	0.451570	0.016*	
C5B	0.6031 (13)	0.3920 (2)	0.5365 (4)	0.0124 (13)	
H5B	0.720376	0.377801	0.576377	0.015*	
C6B	0.7416 (12)	0.4265 (2)	0.4802 (4)	0.0108 (12)	
C7B	0.4260 (12)	0.4256 (2)	0.6605 (4)	0.0132 (12)	
C8B	0.1993 (14)	0.4486 (2)	0.6962 (4)	0.0170 (15)	
H8B1	0.226562	0.482655	0.701378	0.020*	
H8B2	0.058520	0.443594	0.658944	0.020*	
C9B	0.1433 (13)	0.4279 (2)	0.7799 (4)	0.0161 (14)	
H9B1	0.101691	0.394475	0.773782	0.019*	
H9B2	0.290168	0.430251	0.815254	0.019*	
N1B	0.8549 (11)	0.3035 (2)	0.4558 (4)	0.0153 (12)	
H1B	0.890076	0.298643	0.507852	0.018*	
N2B	0.8732 (12)	0.3020 (2)	0.3245 (4)	0.0191 (13)	
H2B1	0.922244	0.296079	0.274052	0.023*	
N3B	0.4064 (11)	0.41495 (19)	0.5812 (4)	0.0132 (11)	
H3B1	0.270436	0.421988	0.555051	0.016*	
N4B	−0.0610 (11)	0.45244 (19)	0.8192 (4)	0.0168 (13)	
H4B3	−0.084777	0.440727	0.870501	0.025*	
H4B4	−0.199126	0.448348	0.788818	0.025*	
H4B5	−0.025994	0.483447	0.822605	0.025*	
O1B	0.9666 (9)	0.42784 (18)	0.4835 (3)	0.0192 (11)	
O2B	0.6074 (9)	0.45059 (16)	0.4326 (3)	0.0165 (10)	
O3B	0.6095 (10)	0.41818 (19)	0.7023 (3)	0.0242 (12)	
C1C	0.1463 (13)	0.6691 (2)	0.3195 (4)	0.0128 (13)	
C2C	0.1565 (13)	0.6706 (2)	0.4018 (5)	0.0141 (14)	
H2C	0.047334	0.655617	0.438715	0.017*	
C3C	0.4622 (15)	0.7138 (2)	0.3545 (5)	0.0213 (17)	
H3C	0.600378	0.733847	0.351904	0.026*	
C4C	−0.0249 (14)	0.6453 (2)	0.2634 (4)	0.0153 (14)	
H4C1	−0.098117	0.668765	0.226096	0.018*	
H4C2	−0.158958	0.631385	0.296104	0.018*	
C5C	0.0961 (13)	0.6068 (2)	0.2117 (4)	0.0123 (13)	
H5C	0.213853	0.621079	0.171984	0.015*	
C6C	0.2323 (12)	0.5721 (2)	0.2681 (4)	0.0122 (12)	
C7C	−0.0780 (14)	0.5710 (2)	0.0880 (4)	0.0164 (13)	
C8C	−0.2973 (12)	0.5473 (2)	0.0521 (4)	0.0165 (14)	
H8C1	−0.439478	0.551611	0.088965	0.020*	
H8C2	−0.265369	0.513438	0.046878	0.020*	
C9C	−0.3547 (13)	0.5675 (2)	−0.0313 (4)	0.0166 (14)	
H9C1	−0.395614	0.601055	−0.025275	0.020*	
H9C2	−0.208148	0.565132	−0.066711	0.020*	
N1C	0.3415 (11)	0.69653 (19)	0.2906 (4)	0.0155 (12)	
H1C	0.377680	0.701450	0.238707	0.019*	
N2C	0.3564 (12)	0.6981 (2)	0.4230 (4)	0.0190 (13)	
H2C1	0.405214	0.704179	0.473316	0.023*	
N3C	−0.0987 (11)	0.58344 (19)	0.1671 (4)	0.0139 (12)	
H3C1	−0.236078	0.577190	0.193104	0.017*	
N4C	−0.5595 (11)	0.5433 (2)	−0.0706 (4)	0.0163 (12)	
H4C3	−0.574463	0.553281	−0.123423	0.024*	
H4C4	−0.699998	0.549560	−0.042776	0.024*	
H4C5	−0.531174	0.511977	−0.070125	0.024*	
O1C	0.1031 (9)	0.54835 (16)	0.3148 (3)	0.0164 (11)	
O2C	0.4603 (9)	0.57167 (18)	0.2645 (3)	0.0199 (11)	
O3C	0.1097 (10)	0.57738 (19)	0.0469 (3)	0.0266 (13)	
C1D	1.1196 (13)	0.6667 (2)	0.8202 (4)	0.0128 (13)	
C2D	1.1380 (14)	0.6656 (2)	0.9031 (5)	0.0152 (14)	
H2D	1.029951	0.650016	0.939607	0.018*	
C3D	1.4456 (13)	0.7071 (3)	0.8575 (4)	0.0186 (15)	
H3D	1.589908	0.725561	0.855240	0.022*	
C4D	0.9321 (13)	0.6473 (2)	0.7643 (4)	0.0139 (14)	
H4D1	0.871802	0.672581	0.728162	0.017*	
H4D2	0.792050	0.636108	0.797437	0.017*	
C5D	1.0255 (13)	0.6063 (2)	0.7097 (4)	0.0137 (13)	
H5D	1.145552	0.618184	0.668399	0.016*	
C6D	1.1492 (13)	0.5708 (2)	0.7664 (4)	0.0119 (12)	
C7D	0.7994 (13)	0.5780 (2)	0.5878 (4)	0.0153 (13)	
C8D	0.5646 (14)	0.5546 (3)	0.5606 (4)	0.0200 (15)	
H8D1	0.430611	0.564434	0.597742	0.024*	
H8D2	0.583439	0.520389	0.565927	0.024*	
C9D	0.4929 (14)	0.5662 (2)	0.4723 (4)	0.0202 (15)	
H9D1	0.326172	0.554301	0.461277	0.024*	
H9D2	0.490527	0.600568	0.465047	0.024*	
N1D	1.3140 (11)	0.69293 (19)	0.7932 (4)	0.0154 (13)	
H1D	1.346639	0.699361	0.741551	0.019*	
N2D	1.3405 (12)	0.6911 (2)	0.9248 (4)	0.0183 (13)	
H2D1	1.391867	0.695914	0.975300	0.022*	
N3D	0.8159 (10)	0.58597 (19)	0.6684 (4)	0.0130 (12)	
H3D1	0.689357	0.578279	0.698960	0.016*	
N4D	0.6663 (10)	0.54536 (18)	0.4130 (3)	0.0136 (11)	
H4D3	0.621520	0.553349	0.361033	0.020*	
H4D4	0.664180	0.513717	0.418170	0.020*	
H4D5	0.819867	0.556165	0.423240	0.020*	
O1D	1.3782 (10)	0.56976 (17)	0.7678 (3)	0.0182 (11)	
O2D	1.0098 (10)	0.54720 (16)	0.8113 (3)	0.0183 (11)	
O3D	0.9634 (9)	0.58748 (18)	0.5381 (3)	0.0203 (11)	
Br1	1.56379 (13)	0.70170 (2)	1.10573 (4)	0.01930 (16)	
Br2	1.05535 (14)	0.29847 (2)	0.64419 (5)	0.0199 (2)	
Br3	0.52414 (14)	0.70397 (2)	0.60864 (4)	0.02070 (17)	
Br4	1.04519 (14)	0.29020 (2)	0.13994 (5)	0.0208 (2)	

### 5-[(2S)-2-(3-Azaniumylpropanamido)-2-carboxylatoethyl]-1H-imidazol-3-ium bromide Atomic displacement parameters (Å^2^)

**Table:**

	*U*^11^	*U*^22^	*U*^33^	*U*^12^	*U*^13^	*U*^23^
C1A	0.016 (3)	0.009 (3)	0.012 (3)	−0.003 (2)	−0.002 (3)	−0.001 (2)
C2A	0.016 (3)	0.016 (3)	0.021 (4)	−0.004 (3)	−0.001 (3)	−0.007 (3)
C3A	0.023 (4)	0.011 (3)	0.013 (3)	−0.001 (3)	−0.001 (3)	−0.001 (2)
C4A	0.016 (3)	0.012 (3)	0.017 (4)	−0.003 (2)	0.006 (3)	−0.003 (2)
C5A	0.020 (3)	0.010 (3)	0.011 (3)	0.001 (2)	0.000 (3)	−0.001 (2)
C6A	0.019 (3)	0.010 (3)	0.006 (3)	−0.002 (2)	−0.004 (3)	−0.003 (2)
C7A	0.023 (3)	0.012 (3)	0.009 (3)	0.000 (3)	−0.002 (3)	0.000 (2)
C8A	0.020 (3)	0.027 (3)	0.008 (3)	0.007 (3)	−0.001 (3)	0.000 (3)
C9A	0.022 (3)	0.024 (3)	0.011 (3)	−0.005 (3)	0.002 (3)	−0.005 (3)
N1A	0.017 (3)	0.013 (3)	0.012 (3)	−0.002 (2)	−0.002 (2)	−0.002 (2)
N2A	0.023 (3)	0.018 (3)	0.016 (3)	−0.003 (3)	0.006 (3)	−0.002 (3)
N3A	0.020 (3)	0.015 (3)	0.009 (3)	0.003 (2)	−0.002 (2)	0.000 (2)
N4A	0.013 (3)	0.013 (2)	0.019 (3)	0.000 (2)	0.001 (2)	0.002 (2)
O1A	0.019 (2)	0.024 (3)	0.011 (3)	−0.004 (2)	0.000 (2)	0.002 (2)
O2A	0.021 (2)	0.014 (2)	0.010 (2)	−0.0001 (19)	−0.005 (2)	0.0024 (17)
O3A	0.019 (3)	0.030 (3)	0.014 (3)	0.005 (2)	−0.006 (2)	−0.008 (2)
C1B	0.017 (3)	0.009 (3)	0.014 (3)	0.001 (2)	−0.001 (3)	−0.002 (2)
C2B	0.028 (4)	0.010 (3)	0.017 (3)	−0.001 (3)	0.004 (3)	0.003 (3)
C3B	0.022 (3)	0.016 (3)	0.021 (4)	0.002 (3)	0.006 (3)	−0.006 (3)
C4B	0.014 (3)	0.012 (3)	0.015 (3)	−0.001 (2)	0.001 (3)	−0.003 (2)
C5B	0.016 (3)	0.014 (3)	0.007 (3)	0.004 (2)	−0.002 (3)	−0.001 (2)
C6B	0.0133 (15)	0.0100 (15)	0.0092 (16)	0.0000 (11)	−0.0006 (11)	−0.0007 (11)
C7B	0.011 (3)	0.011 (3)	0.017 (3)	0.000 (2)	−0.003 (3)	−0.005 (2)
C8B	0.024 (4)	0.019 (3)	0.008 (3)	0.004 (3)	0.004 (3)	0.002 (2)
C9B	0.017 (3)	0.019 (3)	0.013 (3)	0.002 (3)	−0.001 (3)	0.003 (3)
N1B	0.023 (3)	0.012 (3)	0.011 (3)	0.002 (2)	−0.002 (2)	−0.003 (2)
N2B	0.020 (3)	0.019 (3)	0.019 (3)	−0.004 (2)	0.003 (3)	−0.008 (3)
N3B	0.013 (3)	0.017 (3)	0.010 (3)	0.003 (2)	−0.004 (2)	−0.003 (2)
N4B	0.021 (3)	0.014 (3)	0.016 (3)	−0.007 (2)	0.005 (3)	−0.002 (2)
O1B	0.020 (2)	0.026 (3)	0.011 (3)	−0.001 (2)	−0.002 (2)	0.005 (2)
O2B	0.020 (2)	0.014 (2)	0.015 (2)	0.0009 (19)	−0.004 (2)	0.0021 (18)
O3B	0.018 (3)	0.039 (3)	0.016 (3)	0.005 (2)	−0.008 (2)	−0.010 (2)
C1C	0.019 (3)	0.005 (3)	0.014 (3)	0.002 (2)	0.001 (3)	−0.002 (2)
C2C	0.017 (3)	0.013 (3)	0.013 (3)	0.001 (3)	−0.002 (3)	−0.002 (2)
C3C	0.027 (4)	0.015 (4)	0.022 (4)	−0.002 (3)	0.002 (3)	−0.002 (3)
C4C	0.023 (4)	0.015 (3)	0.008 (3)	0.002 (3)	−0.004 (3)	0.000 (2)
C5C	0.015 (3)	0.014 (3)	0.008 (3)	−0.006 (2)	−0.003 (3)	−0.002 (2)
C6C	0.0149 (16)	0.0114 (15)	0.0105 (16)	0.0001 (11)	−0.0001 (11)	−0.0008 (11)
C7C	0.021 (3)	0.013 (3)	0.015 (3)	−0.003 (3)	−0.003 (3)	−0.001 (2)
C8C	0.011 (3)	0.018 (3)	0.021 (3)	−0.003 (3)	−0.007 (3)	0.000 (3)
C9C	0.013 (3)	0.014 (3)	0.023 (4)	−0.001 (3)	−0.005 (3)	0.002 (3)
N1C	0.019 (3)	0.009 (3)	0.018 (3)	−0.001 (2)	0.000 (2)	0.002 (2)
N2C	0.025 (3)	0.017 (3)	0.015 (3)	−0.002 (3)	−0.006 (3)	−0.007 (2)
N3C	0.019 (3)	0.014 (3)	0.009 (3)	−0.004 (2)	0.000 (2)	0.001 (2)
N4C	0.017 (3)	0.015 (3)	0.017 (3)	0.001 (2)	−0.002 (2)	−0.001 (2)
O1C	0.020 (2)	0.012 (2)	0.017 (3)	−0.0008 (19)	0.000 (2)	0.0004 (18)
O2C	0.020 (3)	0.026 (3)	0.013 (3)	−0.001 (2)	−0.004 (2)	0.004 (2)
O3C	0.021 (3)	0.035 (3)	0.024 (3)	−0.008 (2)	0.005 (2)	−0.010 (2)
C1D	0.015 (3)	0.008 (3)	0.015 (3)	0.003 (2)	−0.004 (3)	−0.001 (2)
C2D	0.018 (3)	0.014 (3)	0.014 (3)	0.001 (3)	−0.003 (3)	−0.001 (3)
C3D	0.020 (3)	0.015 (3)	0.022 (4)	0.005 (3)	−0.002 (3)	−0.005 (3)
C4D	0.020 (3)	0.011 (3)	0.011 (3)	0.003 (3)	−0.009 (3)	−0.001 (2)
C5D	0.021 (3)	0.010 (3)	0.009 (3)	−0.001 (2)	−0.004 (3)	0.001 (2)
C6D	0.018 (3)	0.012 (3)	0.006 (3)	0.003 (2)	−0.002 (3)	−0.004 (2)
C7D	0.017 (3)	0.019 (3)	0.010 (3)	−0.003 (3)	0.000 (3)	0.001 (2)
C8D	0.016 (3)	0.026 (4)	0.018 (3)	−0.006 (3)	−0.003 (3)	−0.005 (3)
C9D	0.017 (3)	0.021 (3)	0.022 (4)	0.007 (3)	−0.001 (3)	0.001 (3)
N1D	0.017 (3)	0.014 (3)	0.015 (3)	−0.001 (2)	0.000 (2)	−0.001 (2)
N2D	0.021 (3)	0.017 (3)	0.016 (3)	0.004 (2)	−0.009 (3)	−0.004 (2)
N3D	0.012 (3)	0.017 (3)	0.010 (3)	−0.005 (2)	−0.003 (2)	0.001 (2)
N4D	0.010 (2)	0.014 (2)	0.017 (3)	0.001 (2)	−0.002 (2)	0.001 (2)
O1D	0.022 (3)	0.022 (2)	0.012 (3)	0.006 (2)	−0.005 (2)	0.003 (2)
O2D	0.030 (3)	0.010 (2)	0.015 (2)	−0.004 (2)	0.004 (2)	0.0003 (17)
O3D	0.011 (2)	0.032 (3)	0.018 (3)	−0.006 (2)	0.001 (2)	−0.007 (2)
Br1	0.0218 (3)	0.0225 (3)	0.0136 (3)	−0.0042 (4)	−0.0046 (3)	0.0042 (3)
Br2	0.0230 (4)	0.0231 (4)	0.0135 (4)	0.0070 (3)	0.0044 (3)	0.0039 (3)
Br3	0.0248 (3)	0.0239 (3)	0.0134 (4)	−0.0033 (4)	−0.0029 (3)	0.0037 (3)
Br4	0.0231 (4)	0.0244 (5)	0.0148 (4)	0.0011 (3)	0.0042 (3)	0.0043 (3)

### 5-[(2S)-2-(3-Azaniumylpropanamido)-2-carboxylatoethyl]-1H-imidazol-3-ium bromide Geometric parameters (Å, º)

**Table:**

C1A—C3A	1.362 (10)	C1C—C2C	1.342 (10)
C1A—N1A	1.386 (9)	C1C—N1C	1.405 (9)
C1A—C4A	1.492 (9)	C1C—C4C	1.474 (11)
C2A—N2A	1.300 (10)	C2C—N2C	1.390 (10)
C2A—N1A	1.330 (9)	C2C—H2C	0.9500
C2A—H2A	0.9500	C3C—N1C	1.327 (10)
C3A—N2A	1.381 (9)	C3C—N2C	1.334 (9)
C3A—H3A	0.9500	C3C—H3C	0.9500
C4A—C5A	1.542 (10)	C4C—C5C	1.538 (9)
C4A—H4A1	0.9900	C4C—H4C1	0.9900
C4A—H4A2	0.9900	C4C—H4C2	0.9900
C5A—N3A	1.462 (8)	C5C—N3C	1.452 (9)
C5A—C6A	1.541 (9)	C5C—C6C	1.544 (10)
C5A—H5A	1.0000	C5C—H5C	1.0000
C6A—O2A	1.242 (8)	C6C—O1C	1.242 (8)
C6A—O1A	1.256 (9)	C6C—O2C	1.247 (8)
C7A—O3A	1.236 (9)	C7C—O3C	1.239 (8)
C7A—N3A	1.330 (8)	C7C—N3C	1.343 (9)
C7A—C8A	1.528 (9)	C7C—C8C	1.495 (10)
C8A—C9A	1.527 (9)	C8C—C9C	1.508 (10)
C8A—H8A1	0.9900	C8C—H8C1	0.9900
C8A—H8A2	0.9900	C8C—H8C2	0.9900
C9A—N4A	1.466 (9)	C9C—N4C	1.465 (9)
C9A—H9A1	0.9900	C9C—H9C1	0.9900
C9A—H9A2	0.9900	C9C—H9C2	0.9900
N1A—H1A	0.8800	N1C—H1C	0.8800
N2A—H2A1	0.8800	N2C—H2C1	0.8800
N3A—H3A1	0.8800	N3C—H3C1	0.8800
N4A—H4A3	0.9100	N4C—H4C3	0.9100
N4A—H4A4	0.9100	N4C—H4C4	0.9100
N4A—H4A5	0.9100	N4C—H4C5	0.9100
C1B—C2B	1.351 (10)	C1D—C2D	1.353 (10)
C1B—N1B	1.380 (10)	C1D—N1D	1.373 (9)
C1B—C4B	1.491 (9)	C1D—C4D	1.480 (11)
C2B—N2B	1.375 (9)	C2D—N2D	1.372 (10)
C2B—H2B	0.9500	C2D—H2D	0.9500
C3B—N2B	1.331 (11)	C3D—N2D	1.321 (9)
C3B—N1B	1.339 (9)	C3D—N1D	1.333 (10)
C3B—H3B	0.9500	C3D—H3D	0.9500
C4B—C5B	1.548 (9)	C4D—C5D	1.558 (9)
C4B—H4B1	0.9900	C4D—H4D1	0.9900
C4B—H4B2	0.9900	C4D—H4D2	0.9900
C5B—N3B	1.455 (8)	C5D—N3D	1.450 (9)
C5B—C6B	1.546 (8)	C5D—C6D	1.531 (10)
C5B—H5B	1.0000	C5D—H5D	1.0000
C6B—O1B	1.232 (8)	C6D—O1D	1.252 (9)
C6B—O2B	1.270 (8)	C6D—O2D	1.253 (8)
C7B—O3B	1.231 (9)	C7D—O3D	1.239 (8)
C7B—N3B	1.332 (9)	C7D—N3D	1.336 (9)
C7B—C8B	1.519 (9)	C7D—C8D	1.513 (10)
C8B—C9B	1.518 (8)	C8D—C9D	1.528 (11)
C8B—H8B1	0.9900	C8D—H8D1	0.9900
C8B—H8B2	0.9900	C8D—H8D2	0.9900
C9B—N4B	1.465 (8)	C9D—N4D	1.480 (8)
C9B—H9B1	0.9900	C9D—H9D1	0.9900
C9B—H9B2	0.9900	C9D—H9D2	0.9900
N1B—H1B	0.8800	N1D—H1D	0.8800
N2B—H2B1	0.8800	N2D—H2D1	0.8800
N3B—H3B1	0.8800	N3D—H3D1	0.8800
N4B—H4B3	0.9100	N4D—H4D3	0.9100
N4B—H4B4	0.9100	N4D—H4D4	0.9100
N4B—H4B5	0.9100	N4D—H4D5	0.9100
			
C3A—C1A—N1A	106.0 (6)	C2C—C1C—N1C	106.6 (7)
C3A—C1A—C4A	131.0 (7)	C2C—C1C—C4C	131.2 (6)
N1A—C1A—C4A	123.0 (6)	N1C—C1C—C4C	122.2 (6)
N2A—C2A—N1A	109.1 (7)	C1C—C2C—N2C	107.3 (6)
N2A—C2A—H2A	125.5	C1C—C2C—H2C	126.3
N1A—C2A—H2A	125.5	N2C—C2C—H2C	126.3
C1A—C3A—N2A	106.8 (7)	N1C—C3C—N2C	108.3 (7)
C1A—C3A—H3A	126.6	N1C—C3C—H3C	125.8
N2A—C3A—H3A	126.6	N2C—C3C—H3C	125.8
C1A—C4A—C5A	113.9 (6)	C1C—C4C—C5C	113.5 (6)
C1A—C4A—H4A1	108.8	C1C—C4C—H4C1	108.9
C5A—C4A—H4A1	108.8	C5C—C4C—H4C1	108.9
C1A—C4A—H4A2	108.8	C1C—C4C—H4C2	108.9
C5A—C4A—H4A2	108.8	C5C—C4C—H4C2	108.9
H4A1—C4A—H4A2	107.7	H4C1—C4C—H4C2	107.7
N3A—C5A—C6A	109.8 (5)	N3C—C5C—C4C	106.8 (6)
N3A—C5A—C4A	107.2 (6)	N3C—C5C—C6C	110.8 (5)
C6A—C5A—C4A	107.6 (5)	C4C—C5C—C6C	110.0 (6)
N3A—C5A—H5A	110.7	N3C—C5C—H5C	109.7
C6A—C5A—H5A	110.7	C4C—C5C—H5C	109.7
C4A—C5A—H5A	110.7	C6C—C5C—H5C	109.7
O2A—C6A—O1A	125.8 (6)	O1C—C6C—O2C	126.2 (7)
O2A—C6A—C5A	117.2 (6)	O1C—C6C—C5C	116.3 (6)
O1A—C6A—C5A	116.9 (6)	O2C—C6C—C5C	117.4 (6)
O3A—C7A—N3A	124.1 (7)	O3C—C7C—N3C	123.4 (7)
O3A—C7A—C8A	121.2 (6)	O3C—C7C—C8C	121.3 (6)
N3A—C7A—C8A	114.7 (6)	N3C—C7C—C8C	115.3 (6)
C9A—C8A—C7A	112.4 (6)	C7C—C8C—C9C	110.2 (6)
C9A—C8A—H8A1	109.1	C7C—C8C—H8C1	109.6
C7A—C8A—H8A1	109.1	C9C—C8C—H8C1	109.6
C9A—C8A—H8A2	109.1	C7C—C8C—H8C2	109.6
C7A—C8A—H8A2	109.1	C9C—C8C—H8C2	109.6
H8A1—C8A—H8A2	107.9	H8C1—C8C—H8C2	108.1
N4A—C9A—C8A	112.0 (6)	N4C—C9C—C8C	111.8 (5)
N4A—C9A—H9A1	109.2	N4C—C9C—H9C1	109.3
C8A—C9A—H9A1	109.2	C8C—C9C—H9C1	109.3
N4A—C9A—H9A2	109.2	N4C—C9C—H9C2	109.3
C8A—C9A—H9A2	109.2	C8C—C9C—H9C2	109.3
H9A1—C9A—H9A2	107.9	H9C1—C9C—H9C2	107.9
C2A—N1A—C1A	108.7 (6)	C3C—N1C—C1C	108.8 (6)
C2A—N1A—H1A	125.6	C3C—N1C—H1C	125.6
C1A—N1A—H1A	125.6	C1C—N1C—H1C	125.6
C2A—N2A—C3A	109.5 (6)	C3C—N2C—C2C	108.9 (7)
C2A—N2A—H2A1	125.3	C3C—N2C—H2C1	125.5
C3A—N2A—H2A1	125.3	C2C—N2C—H2C1	125.5
C7A—N3A—C5A	124.6 (7)	C7C—N3C—C5C	122.7 (6)
C7A—N3A—H3A1	117.7	C7C—N3C—H3C1	118.6
C5A—N3A—H3A1	117.7	C5C—N3C—H3C1	118.6
C9A—N4A—H4A3	109.5	C9C—N4C—H4C3	109.5
C9A—N4A—H4A4	109.5	C9C—N4C—H4C4	109.5
H4A3—N4A—H4A4	109.5	H4C3—N4C—H4C4	109.5
C9A—N4A—H4A5	109.5	C9C—N4C—H4C5	109.5
H4A3—N4A—H4A5	109.5	H4C3—N4C—H4C5	109.5
H4A4—N4A—H4A5	109.5	H4C4—N4C—H4C5	109.5
C2B—C1B—N1B	106.3 (6)	C2D—C1D—N1D	106.0 (7)
C2B—C1B—C4B	131.3 (7)	C2D—C1D—C4D	131.0 (6)
N1B—C1B—C4B	122.4 (6)	N1D—C1D—C4D	123.0 (6)
C1B—C2B—N2B	107.1 (7)	C1D—C2D—N2D	107.7 (6)
C1B—C2B—H2B	126.5	C1D—C2D—H2D	126.1
N2B—C2B—H2B	126.5	N2D—C2D—H2D	126.1
N2B—C3B—N1B	106.7 (7)	N2D—C3D—N1D	108.1 (6)
N2B—C3B—H3B	126.6	N2D—C3D—H3D	125.9
N1B—C3B—H3B	126.6	N1D—C3D—H3D	125.9
C1B—C4B—C5B	113.1 (6)	C1D—C4D—C5D	114.1 (6)
C1B—C4B—H4B1	109.0	C1D—C4D—H4D1	108.7
C5B—C4B—H4B1	109.0	C5D—C4D—H4D1	108.7
C1B—C4B—H4B2	109.0	C1D—C4D—H4D2	108.7
C5B—C4B—H4B2	109.0	C5D—C4D—H4D2	108.7
H4B1—C4B—H4B2	107.8	H4D1—C4D—H4D2	107.6
N3B—C5B—C6B	111.8 (5)	N3D—C5D—C6D	111.2 (5)
N3B—C5B—C4B	106.9 (5)	N3D—C5D—C4D	107.9 (6)
C6B—C5B—C4B	110.0 (5)	C6D—C5D—C4D	107.5 (6)
N3B—C5B—H5B	109.4	N3D—C5D—H5D	110.0
C6B—C5B—H5B	109.4	C6D—C5D—H5D	110.0
C4B—C5B—H5B	109.4	C4D—C5D—H5D	110.0
O1B—C6B—O2B	125.9 (6)	O1D—C6D—O2D	125.8 (7)
O1B—C6B—C5B	118.9 (6)	O1D—C6D—C5D	117.9 (6)
O2B—C6B—C5B	115.2 (6)	O2D—C6D—C5D	116.2 (6)
O3B—C7B—N3B	124.2 (6)	O3D—C7D—N3D	123.8 (7)
O3B—C7B—C8B	121.9 (6)	O3D—C7D—C8D	121.3 (6)
N3B—C7B—C8B	113.9 (6)	N3D—C7D—C8D	114.9 (6)
C9B—C8B—C7B	109.8 (6)	C7D—C8D—C9D	113.4 (6)
C9B—C8B—H8B1	109.7	C7D—C8D—H8D1	108.9
C7B—C8B—H8B1	109.7	C9D—C8D—H8D1	108.9
C9B—C8B—H8B2	109.7	C7D—C8D—H8D2	108.9
C7B—C8B—H8B2	109.7	C9D—C8D—H8D2	108.9
H8B1—C8B—H8B2	108.2	H8D1—C8D—H8D2	107.7
N4B—C9B—C8B	111.1 (6)	N4D—C9D—C8D	111.3 (6)
N4B—C9B—H9B1	109.4	N4D—C9D—H9D1	109.4
C8B—C9B—H9B1	109.4	C8D—C9D—H9D1	109.4
N4B—C9B—H9B2	109.4	N4D—C9D—H9D2	109.4
C8B—C9B—H9B2	109.4	C8D—C9D—H9D2	109.4
H9B1—C9B—H9B2	108.0	H9D1—C9D—H9D2	108.0
C3B—N1B—C1B	109.9 (6)	C3D—N1D—C1D	109.4 (6)
C3B—N1B—H1B	125.0	C3D—N1D—H1D	125.3
C1B—N1B—H1B	125.0	C1D—N1D—H1D	125.3
C3B—N2B—C2B	109.9 (6)	C3D—N2D—C2D	108.8 (7)
C3B—N2B—H2B1	125.0	C3D—N2D—H2D1	125.6
C2B—N2B—H2B1	125.0	C2D—N2D—H2D1	125.6
C7B—N3B—C5B	122.0 (6)	C7D—N3D—C5D	125.3 (6)
C7B—N3B—H3B1	119.0	C7D—N3D—H3D1	117.3
C5B—N3B—H3B1	119.0	C5D—N3D—H3D1	117.4
C9B—N4B—H4B3	109.5	C9D—N4D—H4D3	109.5
C9B—N4B—H4B4	109.5	C9D—N4D—H4D4	109.5
H4B3—N4B—H4B4	109.5	H4D3—N4D—H4D4	109.5
C9B—N4B—H4B5	109.5	C9D—N4D—H4D5	109.5
H4B3—N4B—H4B5	109.5	H4D3—N4D—H4D5	109.5
H4B4—N4B—H4B5	109.5	H4D4—N4D—H4D5	109.5
			
N1A—C1A—C3A—N2A	−0.1 (7)	N1C—C1C—C2C—N2C	−0.6 (8)
C4A—C1A—C3A—N2A	−178.7 (7)	C4C—C1C—C2C—N2C	179.7 (7)
C3A—C1A—C4A—C5A	−111.9 (9)	C2C—C1C—C4C—C5C	−114.8 (8)
N1A—C1A—C4A—C5A	69.7 (8)	N1C—C1C—C4C—C5C	65.5 (8)
C1A—C4A—C5A—N3A	171.1 (6)	C1C—C4C—C5C—N3C	173.7 (6)
C1A—C4A—C5A—C6A	53.0 (8)	C1C—C4C—C5C—C6C	53.4 (7)
N3A—C5A—C6A—O2A	−45.5 (8)	N3C—C5C—C6C—O1C	−51.6 (8)
C4A—C5A—C6A—O2A	70.9 (7)	C4C—C5C—C6C—O1C	66.3 (8)
N3A—C5A—C6A—O1A	138.7 (6)	N3C—C5C—C6C—O2C	130.7 (7)
C4A—C5A—C6A—O1A	−105.0 (7)	C4C—C5C—C6C—O2C	−111.4 (7)
O3A—C7A—C8A—C9A	−32.0 (9)	O3C—C7C—C8C—C9C	−46.7 (9)
N3A—C7A—C8A—C9A	151.1 (6)	N3C—C7C—C8C—C9C	134.8 (6)
C7A—C8A—C9A—N4A	69.9 (8)	C7C—C8C—C9C—N4C	176.5 (5)
N2A—C2A—N1A—C1A	0.4 (8)	N2C—C3C—N1C—C1C	1.1 (8)
C3A—C1A—N1A—C2A	−0.2 (7)	C2C—C1C—N1C—C3C	−0.3 (8)
C4A—C1A—N1A—C2A	178.6 (6)	C4C—C1C—N1C—C3C	179.4 (6)
N1A—C2A—N2A—C3A	−0.5 (8)	N1C—C3C—N2C—C2C	−1.4 (8)
C1A—C3A—N2A—C2A	0.3 (8)	C1C—C2C—N2C—C3C	1.2 (8)
O3A—C7A—N3A—C5A	0.1 (10)	O3C—C7C—N3C—C5C	1.6 (11)
C8A—C7A—N3A—C5A	176.9 (6)	C8C—C7C—N3C—C5C	−179.9 (6)
C6A—C5A—N3A—C7A	−112.3 (7)	C4C—C5C—N3C—C7C	137.8 (6)
C4A—C5A—N3A—C7A	131.0 (7)	C6C—C5C—N3C—C7C	−102.4 (7)
N1B—C1B—C2B—N2B	−0.2 (8)	N1D—C1D—C2D—N2D	0.2 (8)
C4B—C1B—C2B—N2B	179.6 (7)	C4D—C1D—C2D—N2D	−176.8 (7)
C2B—C1B—C4B—C5B	−114.5 (9)	C2D—C1D—C4D—C5D	−112.1 (8)
N1B—C1B—C4B—C5B	65.3 (8)	N1D—C1D—C4D—C5D	71.2 (8)
C1B—C4B—C5B—N3B	173.6 (6)	C1D—C4D—C5D—N3D	170.9 (6)
C1B—C4B—C5B—C6B	52.1 (8)	C1D—C4D—C5D—C6D	50.8 (7)
N3B—C5B—C6B—O1B	130.1 (7)	N3D—C5D—C6D—O1D	138.7 (6)
C4B—C5B—C6B—O1B	−111.3 (7)	C4D—C5D—C6D—O1D	−103.3 (7)
N3B—C5B—C6B—O2B	−51.5 (8)	N3D—C5D—C6D—O2D	−45.6 (8)
C4B—C5B—C6B—O2B	67.1 (7)	C4D—C5D—C6D—O2D	72.4 (7)
O3B—C7B—C8B—C9B	−43.6 (9)	O3D—C7D—C8D—C9D	−29.2 (10)
N3B—C7B—C8B—C9B	137.0 (6)	N3D—C7D—C8D—C9D	152.5 (6)
C7B—C8B—C9B—N4B	174.7 (6)	C7D—C8D—C9D—N4D	68.2 (8)
N2B—C3B—N1B—C1B	0.7 (8)	N2D—C3D—N1D—C1D	−0.4 (8)
C2B—C1B—N1B—C3B	−0.3 (8)	C2D—C1D—N1D—C3D	0.1 (8)
C4B—C1B—N1B—C3B	179.9 (6)	C4D—C1D—N1D—C3D	177.5 (6)
N1B—C3B—N2B—C2B	−0.8 (8)	N1D—C3D—N2D—C2D	0.6 (8)
C1B—C2B—N2B—C3B	0.6 (8)	C1D—C2D—N2D—C3D	−0.5 (8)
O3B—C7B—N3B—C5B	1.6 (10)	O3D—C7D—N3D—C5D	−0.5 (11)
C8B—C7B—N3B—C5B	−179.0 (5)	C8D—C7D—N3D—C5D	177.7 (6)
C6B—C5B—N3B—C7B	−104.2 (7)	C6D—C5D—N3D—C7D	−113.2 (7)
C4B—C5B—N3B—C7B	135.4 (6)	C4D—C5D—N3D—C7D	129.1 (7)

### 5-[(2S)-2-(3-Azaniumylpropanamido)-2-carboxylatoethyl]-1H-imidazol-3-ium bromide Hydrogen-bond geometry (Å, º)

**Table:**

*D*—H···*A*	*D*—H	H···*A*	*D*···*A*	*D*—H···*A*
N1*A*—H1*A*···Br4^i^	0.88	2.39	3.242 (7)	162
N2*A*—H2*A*1···Br2^ii^	0.88	2.33	3.205 (6)	171
N3*A*—H3*A*1···O1*A*^i^	0.88	2.04	2.918 (9)	172
N4*A*—H4*A*3···O1*B*^iii^	0.91	1.88	2.771 (7)	167
N4*A*—H4*A*4···O1*C*^i^	0.91	1.84	2.744 (7)	171
N4*A*—H4*A*5···O3*A*	0.91	2.23	2.887 (7)	129
N4*A*—H4*A*5···O2*B*^i^	0.91	2.36	2.875 (8)	116
C2*A*—H2*A*···Br1^iv^	0.95	2.71	3.651 (8)	170
C4*A*—H4*A*1···Br4^iii^	0.99	2.77	3.678 (6)	153
C9*A*—H9*A*1···O3*A*^i^	0.99	2.54	3.156 (10)	120
N1*B*—H1*B*···Br2	0.88	2.40	3.261 (7)	167
N2*B*—H2*B*1···Br4	0.88	2.29	3.167 (6)	173
N3*B*—H3*B*1···O1*B*^i^	0.88	2.04	2.907 (8)	170
N4*B*—H4*B*3···O1*A*^v^	0.91	1.86	2.764 (7)	171
N4*B*—H4*B*4···O3*B*^i^	0.91	1.96	2.798 (8)	153
N4*B*—H4*B*5···O2*D*^i^	0.91	1.85	2.744 (7)	169
C2*B*—H2*B*···O3*A*^vi^	0.95	2.58	3.425 (10)	149
C3*B*—H3*B*···Br3^iv^	0.95	2.64	3.587 (8)	171
C4*B*—H4*B*1···Br2^i^	0.99	2.93	3.836 (6)	152
C5*B*—H5*B*···Br2	1.00	3.12	4.046 (7)	155
C8*B*—H8*B*2···O3*B*^i^	0.99	2.66	3.341 (9)	127
C9*B*—H9*B*2···O2*A*^vii^	0.99	2.38	3.307 (9)	156
N1*C*—H1*C*···Br1^ii^	0.88	2.39	3.251 (6)	165
N2*C*—H2*C*1···Br3	0.88	2.30	3.165 (7)	168
N3*C*—H3*C*1···O2*C*^i^	0.88	2.03	2.905 (7)	171
N4*C*—H4*C*3···O1*D*^viii^	0.91	1.85	2.760 (8)	176
N4*C*—H4*C*4···O3*C*^i^	0.91	1.96	2.808 (7)	154
N4*C*—H4*C*5···O2*A*	0.91	1.84	2.747 (7)	176
C2*C*—H2*C*···O3*D*^i^	0.95	2.58	3.422 (9)	148
C3*C*—H3*C*···Br2^ix^	0.95	2.64	3.583 (8)	172
C4*C*—H4*C*1···Br1^viii^	0.99	2.85	3.776 (8)	155
C9*C*—H9*C*2···O2*D*^ii^	0.99	2.37	3.300 (8)	156
N1*D*—H1*D*···Br3^vi^	0.88	2.38	3.234 (6)	165
N2*D*—H2*D*1···Br1	0.88	2.33	3.204 (7)	173
N3*D*—H3*D*1···O1*D*^i^	0.88	2.05	2.926 (7)	172
N4*D*—H4*D*3···O2*C*	0.91	1.88	2.772 (8)	167
N4*D*—H4*D*4···O2*B*	0.91	1.85	2.751 (7)	171
N4*D*—H4*D*5···O1*C*^vi^	0.91	2.36	2.875 (7)	116
N4*D*—H4*D*5···O3*D*	0.91	2.22	2.871 (8)	128
C3*D*—H3*D*···Br4^x^	0.95	2.72	3.662 (8)	170
C4*D*—H4*D*1···Br3	0.99	2.86	3.746 (7)	149
C9*D*—H9*D*1···O3*D*^i^	0.99	2.53	3.146 (8)	120

Symmetry codes: (i) *x*−1, *y*, *z*; (ii) *x*−1, *y*, *z*−1; (iii) *x*−2, *y*, *z*; (iv) −*x*+2, *y*−1/2, −*z*+1; (v) *x*, *y*, *z*+1; (vi) *x*+1, *y*, *z*; (vii) *x*+1, *y*, *z*+1; (viii) *x*−2, *y*, *z*−1; (ix) −*x*+2, *y*+1/2, −*z*+1; (x) −*x*+3, *y*+1/2, −*z*+1.
